# The Antioxidant Properties of *Salvia verbenaca* Extract Contribute to Its Intestinal Antiinflammatory Effects in Experimental Colitis in Rats

**DOI:** 10.3390/antiox12122071

**Published:** 2023-12-02

**Authors:** Teresa Vezza, Jose Alberto Molina-Tijeras, Alba Rodríguez-Nogales, Jose Garrido-Mesa, María de la Luz Cádiz-Gurrea, Antonio Segura-Carretero, María Reyes González-Tejero, María Elena Rodríguez-Cabezas, Julio Gálvez, Francesca Algieri

**Affiliations:** 1 Department of Pharmacology, Center for Biomedical Research (CIBM), University of Granada, 18071 Granada, Spain; tvezza@ibsgranada.es (T.V.); jalbertomolina@ugr.es (J.A.M.-T.); jose.garrido@oncosur.org (J.G.-M.); jgalvez@ugr.es (J.G.); falgieri@ugr.es (F.A.); 2 Instituto de Investigación Biosanitaria de Granada (ibs. GRANADA), 18012 Granada, Spain; 3 Servicio de Digestivo, Hospital Universitario Virgen de las Nieves, 18014 Granada, Spain; 4 Department of Analytical Chemistry, Faculty of Science, University of Granada, 18071 Granada, Spain; mluzcadiz@ugr.es (M.d.l.L.C.-G.); ansegura@ugr.es (A.S.-C.); 5 Department of Botany, University of Granada, 18071 Granada, Spain; mreyes@ugr.es; 6 Centro de Investigación Biomédica en Red de Enfermedades Hepáticas y Digestivas (CIBEREHD), Instituto de Salud Carlos III, 28029 Madrid, Spain

**Keywords:** inflammatory bowel disease, inflammation, TNBS-induced colitis, medicinal plants, *Salvia verbenaca*

## Abstract

Inflammatory bowel disease (IBD) is a chronic gastrointestinal inflammation with unpredictable symptom fluctuations. While there is no effective cure for IBD, various treatments aim to manage symptoms and improve the quality of life for affected individuals. In recent years, there has been growing interest in the potential benefits of certain natural plants and herbs in the management of IBD. In this regard, this study aimed to evaluate the immunomodulatory and anti-inflammatory effects of a well-characterized extract of *Salvia verbenaca* (*S. verbenaca*) in an experimental model of colitis in rats. Interestingly, the daily administration of *S. verbenaca* (10 and 25 mg/kg) effectively alleviated colitis symptoms, as evidenced by reduced weight/length ratio and colonic damage. Moreover, it reduced oxidative stress markers (MPO and GSH), decreased pro-inflammatory cytokine expression (*Il-6*, *Il-12a*, *Il-1β*, *Il-23*, *Icam-1*, *Mcp-1*, *Cinc-1*), and preserved the integrity of the intestinal barrier (*Villin*, *Muc-2*, *Muc-3*). These effects suggest *S. verbenaca* extract could represent a potential complementary candidate to treat gastrointestinal disorders. Its beneficial actions can be related to its antioxidant properties as well as the downregulation of the immune response, which can result in the improvement in the intestine epithelial barrier.

## 1. Introduction

Inflammatory bowel disease (IBD), which mainly encompasses ulcerative colitis (UC) and Crohn’s disease (CD), refers to idiopathic disorders characterized by spontaneous and chronic relapsing inflammation of the gastrointestinal tract. Although its clinical course is largely unpredictable, IBD is characterized by the existence of alternative periods of exacerbation and remission in the symptoms, which include diarrhea, vomiting, rectal bleeding, and abdominal cramps/discomfort, thus reflecting a situation of epithelial barrier disruption and mucosal ulceration [1,2]. Its etiology is still unclear, although many studies have proposed a prominent role of gut luminal bacteria together with an impaired intestinal barrier function. This triggers an abnormal immune response in IBD patients, thus contributing to the initiation and progression of these intestinal conditions [3]. The altered immune response encompasses the activation of different immune cells (monocytes, neutrophils, T lymphocytes, or macrophages) with overexpression and release of pro-inflammatory mediators, including cytokines such as interleukin (IL)-6, IL-1 β, and tumor necrosis factor-alpha (TNF-α), as well as activated reactive oxygen and nitrogen species, which play a critical role in the development of IBD [4]. 

As there is currently no specific curative treatment for human IBD, different drugs are currently used to target multiple stages of the inflammatory cascade, mainly by using immunosuppressants, glucocorticoids or aminosalicylates, and, more recently, biological agents [5,6]. However, these drugs exhibit limited efficacy for long-term remission and are linked to significant side effects [7]. For this reason, there is a clear demand for new strategies combining efficacy and safety for therapeutic IBD management. 

Natural products have a prominent role as a source of therapeutic agents, especially those from medicinal plants [8,9,10]. Among the Mediterranean area, traditionally recognized as an important source of herbal medicine, *Salvia* seems to be the most interesting. It constitutes an important genus of the *Lamiaceae* family, reputed for its medicinal properties and used in human nutrition and health, particularly in coronary heart diseases and hypertension [11], hepatocirrhosis [12], chronic renal failure [13], and inflammatory disorders [14,15]. Polyphenolic compounds are thought to be responsible, at least in part, for their health effects, but their mechanisms of action and benefit for IBD remain unexplored. For all these reasons, we evaluated the immunomodulatory and anti-inflammatory effects of *S. verbenaca* extract in an acute 2,4,6-Trinitrobenzene Sulfonic Acid (TNBS)-induced experimental colitis, a model that mimics several features of human IBD [16]. We assessed the inflammatory response by determining inflammatory markers such as myeloperoxidase (MPO) activity and cytokines profile. Furthermore, immunomodulatory effects of the extract (0.1–100 μg/mL) were investigated in murine (CMT-93) epithelial cells.

## 2. Materials and Methods

### 2.1. Chemicals and Reagents

The analytical procedures were performed using LC-MS-grade formic acid, acetonitrile, and water for HPLC platforms, which were purchased from Fluka (Sigma-Aldrich, Steinheim, Germany) and Lab-Scan (Gliwice, Sowinskiego, Poland), respectively. For solutions, Milli-Q Milli-pore ultrapure water (Bedford, MA, USA) and methanol VWR Chemicals (Radnor, PA, USA) were used.

For antioxidant capacity assays, the following reagents were provided from the indicated suppliers: hydrochloric acid, sodium hydroxide, TPTZ (2,4,6-tris(2-pyridyl)-s-triazine), acetic acid, and sodium carbonate were acquired from Fluka (Honeywell, NC, USA). Sodium phosphate monobasic and dibasic, AAPH (2,2′-azobis(2-amidinopropane) dihydrochloride), fluorescein, heptahydrate ferrous sulfate, ferric chloride, sodium acetate, Trolox (6-hydroxy-2,5,7,8-tetramethylchroman-2-carboxylic acid), potassium persulfate, ABTS (2,2′-azinobis (3-ethylbenzothiazoline-6-sulphonate)), Folin reagent, and gallic acid were obtained from Sigma-Aldrich (St. Louis, MO, USA). Absolute ethanol was purchased from Riedel-de-Haën (Honeywell, NC, USA). All chemicals, unless otherwise indicated, were purchased from Sigma-Aldrich (Merck Life Science S.L.U., Madrid, Spain).

### 2.2. Plant Material and Preparation of the Extract

Fresh, native *S. verbenaca* plants were collected from urban fields in Granada, Spain (37.19280, −3.599) in April 2016 and authenticated by taxonomist Dr. M.R. González-Tejero, senior researcher of the Botany Department of the University of Granada, Spain. A voucher specimen (No. 62623-1) was deposited in the herbarium of the University of Granada (Granada, Spain). 

The plant extract was prepared as follows: 5 g of ground plant material was mixed with washed sea sand (Panreac Quımica S.A.U., Castellar del Vallés, Spain) and extracted with 30 mL of methanol 50% (*v*/*v*) at 1500 PSI and 80 °C for 10 min in a Dionex ASE200 extraction system (Dionex Corporation, Sunnyvale, CA, USA); this method was already described by Delgado-Torre et al., 2012 [17]. Liquid extract was pooled, and the solvent evaporated under vacuum at 60 °C after two cycles of extraction. Then, land dry extract was stored at −20 °C. Extraction efficiency was 37.64% (*w*/*w*).

### 2.3. Chemical Composition of S. verbenaca Using High-Performance Liquid Chromatography-Mass Spectrometry Condition

For the phytochemical characterization of *S. verbenaca* extract, HPLC-ESI-QTOF analysis was carried out. Firstly, the extract was reconstituted to a concentration of 5 mg/mL in methanol:water (20:80; *v*:*v*). The solution was then vortexed, filtered (0.2 µm), and transferred to an HPLC vial for analysis. In addition, blank samples were prepared to detect contaminants. An HPLC 1290 system coupled to a quadrupole time-of-flight (QTOF) mass spectrometer (G6530C UHD, Agilent Tech., Santa Clara, CA, USA) equipped with a Jet Stream dual ESI interface was used. Chromatographic separation was performed on an analytical C18 column (ACQUITY UPLC BEH Shield RP18 Column, 130 Å, 1.7 µm, 2.1 mm × 150 mm). The mobile phases A and B were acidified water with 0.1% *v*/*v* formic acid and acetonitrile, respectively. The following linear elution gradient was used: 0 min, 100% A; 5 min, 90% A; 18 min 15% A; 24 min, 0% A; 25.50 min, 0% A; 26.50 min, 95% A; 32.50 min, 95% A. The flow rate and the injection volume were 0.4 mL/min and 5 μL, respectively. The MS acquisitions were performed in negative ionization mode and full scan mode (mass-to-charge ratio from 50 to 1200 *m*/*z*). Moreover, the sample was also analyzed in a DDA MS/MS acquisition mode using fixed collision energies of 10.00, 30.00, 60.00 eV. The source parameters were nebulization gas pressure 2 bar, nozzle voltage 500 V, capillary voltage 4 kV, desolvation temperature 350 °C, nebulizer 20 psig, desolvation gas flow resolution 10 L/min, gas temperature 200 °C, and scan duration 1.2 s. The MS raw data were firstly transformed using the MSConverGUI software (https://proteowizard.sourceforge.io/download.html) (accessed on 23 June 2023) and then processed through the open-source software MZmine 2.53. The information obtained was compared with the existing literature to annotate the compounds.

### 2.4. Total Phenolic Content and Antioxidant Capacity Assays

Total phenolic content (TPC) and antioxidant capacity assays were carried out by Folin–Ciocalteu (TPC), FRAP, TEAC, and ORAC, respectively. All assays were performed on a Synergy H1 Monochromator-Based Multi-Mode Microplate reader (Bio-Tek Instruments Inc., Winooski, VT, USA) in accordance with the methodology previously reported [18]. All measurements were made in triplicate.

Moreover, the 2,2-Diphenyl-1-picrylhydrazyl (DPPH) radical scavenging activity of the extract was assessed spectrophotometrically in an MRX+ tc (DYNEX Technologies GmbH, Denkendorf, Germany) by monitoring the disappearance of PPH· at 515 nm, according to a previously described method [19]. The antioxidant activity was expressed as half-maximal inhibitory concentration (IC50) value (in μg/mL), which was obtained by a non-linear regression with a one-phase exponential association equation using GraphPad Prism version 9.0 (GraphPad Software, La Jolla, CA, USA). The antiradical activity was also compared to well-known antioxidants, such as gallic and ascorbic acid.

### 2.5. In Vitro Studies

Murine colonic epithelial CMT-93 cells obtained from the Cell Culture Unit of the University of Granada (Granada, Spain) were maintained in RPMI Medium supplemented with streptomycin (1 mg/mL), penicillin (100 units/mL), L-glutamine (2 mmol/L), and 10% heat-inactivated fetal bovine serum in a humidified 5% CO_2_ atmosphere at 37%. Cells were seeded into 96-well plates at a density of 5 × 10^5^ cells/well, grown until the formation of a monolayer, pre-incubated with different concentrations of the extract ranging from 0.1 to 100 μg/mL for 2 h, and subsequently stimulated with *Escherichia coli* 055:B5 lipopolysaccharide (LPS) at a concentration of 10 μg/mL for 72 h.

LPS-stimulated cells and untreated unstimulated cells were used as positive and negative controls, respectively. After 72 h, measurements of cytokines in cell culture supernatants were assayed by sensitive enzyme-linked immunosorbent assay (ELISA) kits (R&D Systems, Inc., Minneapolis, MN, USA) following the manufacturer’s instructions. 

Finally, the CellTiter 96^®^ AQueous One Solution Cell Proliferation Assay (Promega, Madison, WI, USA) was employed to analyze the effect of the extract on cell viability. Briefly, a small amount of the [3-(4,5-dimethylthiazol-2-yl)-5-(3-carboxymethoxyphenyl)-2-(4-sulfophenyl)-2H-tetrazolium solution was added directly to culture wells and incubated for 1–4 h. The cell culture medium’s absorbance was recorded at 490 nm on an MRX Dynex microplate reader (Dynex Technologies, Chantilly, VA, USA). The cellular viability was determined based on the average absorbance values and compared with those of the untreated control cells. The same experimental design was performed to analyze the RNA expression of various inflammatory mediators. In this case, 2 h after the stimulation with LPS, supernatants were removed, and the cells were collected for RNA extraction.

### 2.6. Animals and Experimental Design

The study was performed in rigorous accordance with the “Guide for the Care and Use of Laboratory Animals” as declared by the US National Institutes of Health (NIH), and all protocols were approved by the Ethics Committee of Laboratory Animals of the University of Granada (Spain) (reference number CEEA-2010-286). Female Wistar rats (180–200 g) derived from Janvier Labs (St Berthevin, France) were housed in Makrolon^®^ cages, kept at constant room temperature, relative humidity (50–80%), and illumination (12/12 h light–dark cycle), and were given unrestricted access to tap water and food. The animals were randomly assigned to five groups (n = 10). Three of them received daily either the corresponding dose of the extract *S. verbenaca* (10 and 25 mg/kg) or dexamethasone (1.2 mg/kg), a synthetic glucocorticoid used as a positive control. Both the extract and dexamethasone were suspended in 1 mL of carboxymethylcellulose and administered by oral gavage. A non-colitic (NC) group and an untreated TNBS control group were also included as reference. These groups received only the vehicle used for administering the test compounds. Colitis was induced in both control and treated groups as indicated in previous reports [20]. In summary, rats underwent an overnight fasting period, were deeply anesthetized using isoflurane (Isoflo^®^, Esteve, Barcelona, Spain), and were administered 10 mg of TNBS dissolved in 0.25 mL of 50% ethanol (*v*/*v*), which was instilled into the colon by using a Teflon cannula inserted 8 cm through the anus. Animals were kept in a head-down position until they gradually recovered from anesthesia, after which they were then returned to their home cages. The remaining animals underwent intracolonic administration of phosphate-buffered saline (0.25 mL) instead of TNBS. Treatments described were administered starting the same day of colitis induction until the day before rats’ sacrifice, which took place 8 days after the induction of the colonic damage. Water and food intake, occurrence of diarrhea, and animal body weights were monitored daily throughout the entire experiment. After mask inhalation of isoflurane, animals were sacrificed. 

Then, the colonic segment was collected aseptically, placed on an ice-cooled plate, longitudinally opened, and thoroughly rinsed to remove its luminal contents with ice-cold saline solution. Subsequently, each specimen was weighed, and its length measured. Colonic damage score (0–10 scale) (Table 1) was macroscopically examined by two independent observers, according to previously reported procedures [20]. 

Subsequently, the collected colonic samples were divided into various longitudinal fragments for biochemical determinations or RNA isolation. Interestingly, the activity of a specific marker of neutrophil infiltration, known as myeloperoxidase (MPO), was assayed in colon homogenates according to the procedures described by Krawisz et al. [21], and the results were expressed as MPO units per gram of wet tissue; one unit of MPO activity was defined as the amount needed to degrade 1 µmol hydrogen peroxide per minute at 25 °C. Similarly, the quantitative measurement of total glutathione content was determined with the enzymatic recycling assay reported by Anderson et al. [22], and the results were expressed as nmol/g wet tissue.

### 2.7. Analysis of Gene Expression by RT-qPCR

Total RNA (from colon or cells) was isolated following the standard Tri-Reagent^®^ protocol and quantified using the Thermo Scientific NanoDrop™ 2000 Spectrophotometer (Thermo Fisher Scientific Inc., Waltham, MA, USA). Afterwards, 2 μg of RNA was reverse transcribed using oligo(dT) primers (Promega, Southampton, UK). Real-time quantitative PCR amplification and detection was performed on optical-grade 48-well plates in an EcoTM Real-Time PCR System (Illumina, San Diego, CA, USA) with 20 ng of cDNA, the KAPA SYBR^®^ FAST qPCR Master Mix (Kapa Biosystems, Inc., Wilmington, MA, USA), and specific primers at their annealing temperature (Ta) (Table 2). The expression of the housekeeping gene glyceraldehyde-3-phosphate dehydrogenase (*Gapdh*) was used to normalize (m)RNA expression. The relative changes in gene expression were calculated using the ∆∆Ct method [23].

### 2.8. Statistics

All results are expressed as the mean ± SEM. Differences between means were tested for statistical significance using a one-way analysis of variance (ANOVA) and post hoc least significance tests. Non-parametric data (score) are expressed as the median (range) and were analyzed using the Kruskal–Wallis test. All statistical analyses were performed with GraphPad Prism version 9.0 (GraphPad Software Inc., La Jolla, CA, USA) with statistical significance set at *p* < 0.05.

## 3. Results 

### 3.1. Chemical Characterization of S. verbenaca

**Figure 1:**
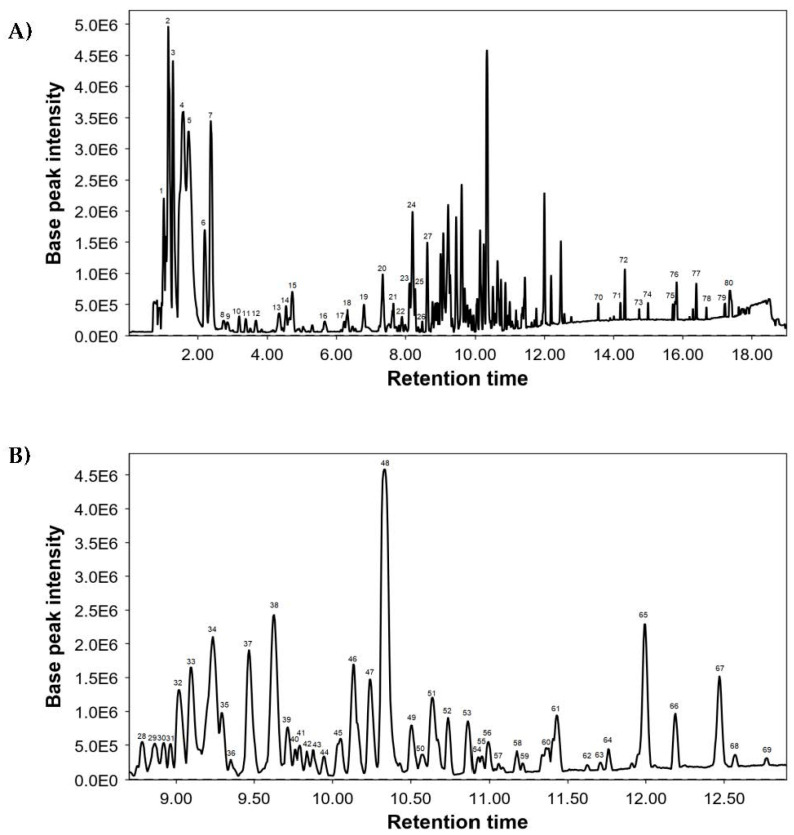
Base peak chromatogram of *S. verbenaca* by UHPL-QTOF: (**A**) full chromatogram and (**B**) retention time from 8.50 to 13 min.

The UHPLC-MS was used for a comprehensive characterization of the chemical composition of *S. verbenaca* extract, and Figure 1 shows its base peak chromatogram (BPC). Identified compounds are included in Table 3, numbered per elution order, and indicate their retention times (RT), experimental *m*/*z*, molecular formula, and proposed compounds. A total of 80 compounds were detected, mainly caffeic acid derivatives and flavonoids.

### 3.2. Antioxidant Capacity of S. verbenaca

The total phenolic content (TPC) of *S. verbenaca* was determined using the Folin–Ciocalteu method, which was 166 ± 4 mg GAE/g of plant extract. Subsequently, the antioxidant potential of this extract was confirmed by FRAP, ORAC, and TEAC assays (Table 4). These assays collectively indicated significant free radical scavenging activity in *S. verbenaca*. Additionally, the extract showed an antiradical effect in the DPPH assay, with an IC50 of 59.9 ± 8.7 μg/mL.

### 3.3. Immunomodulatory Properties of S. verbenaca in Murine Intestinal Epithelial Cells

**Figure 2:**
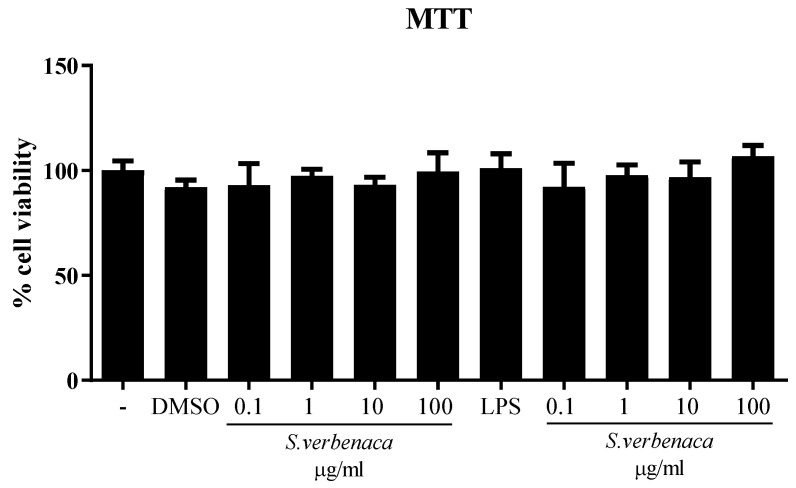
Effects of *S. verbenaca* (0.1–100 μg/mL) on cell viability in CMT-93 cells. Data are expressed as means ± SEM. The experiments were performed three times.

The beneficial effects of *S. verbenaca* extract were evaluated in murine intestinal epithelial cells (CMT-93). Incubation of cells with different concentrations of the extracts (0.1–100 μg/mL) did not affect cell viability and did not induce any inflammatory response (Figure 2). 

**Figure 3:**
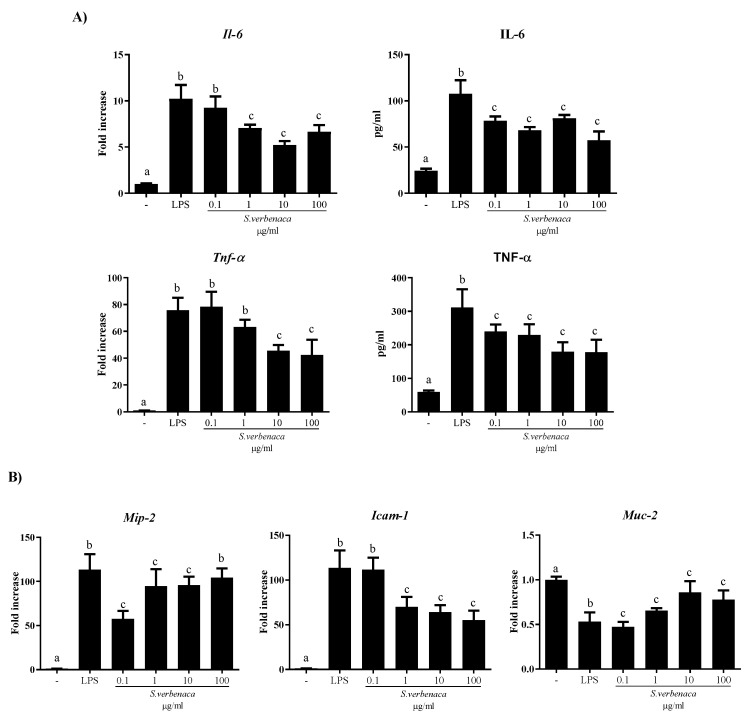
Effects of *S. verbenaca* on (**A**) IL-6 and TNFα expression and release as well as (**B**) *Mip-2*, *Icam-1*, and *Muc-2* gene expression by LPS-stimulated CMT-93 cells. Data are expressed as means ± SEM. Groups with different letters statistically differ (*p* < 0.05).

Stimulation of CMT-93 cells with LPS resulted in pro-inflammatory cytokine (IL-6 and TNF-α) expression and release, quantified by RT-qPCR and ELISA, respectively (Figure 3A). Both cytokines were significantly reduced by the different concentrations of the extract (Figure 3A; *p* < 0.05 vs. LPS).

Similarly, *S. verbenaca* inhibited the increased mRNA expression of *Icam-1* and *Mip-2* in these cells after LPS stimulation (*p* < 0.05 vs. LPS) (Figure 3B). Moreover, the expression of *Muc-2*, a secretory protein crucial for the maintenance of epithelial integrity, was reduced after LPS stimulation of the cells, and this effect was partially reversed with the different doses of the extract (*p* < 0.05 vs. LPS).

### 3.4. Intestinal Anti-Inflammatory Effect of S. verbenaca in TNBS-Induced Colitis in Rats

**Figure 4:**
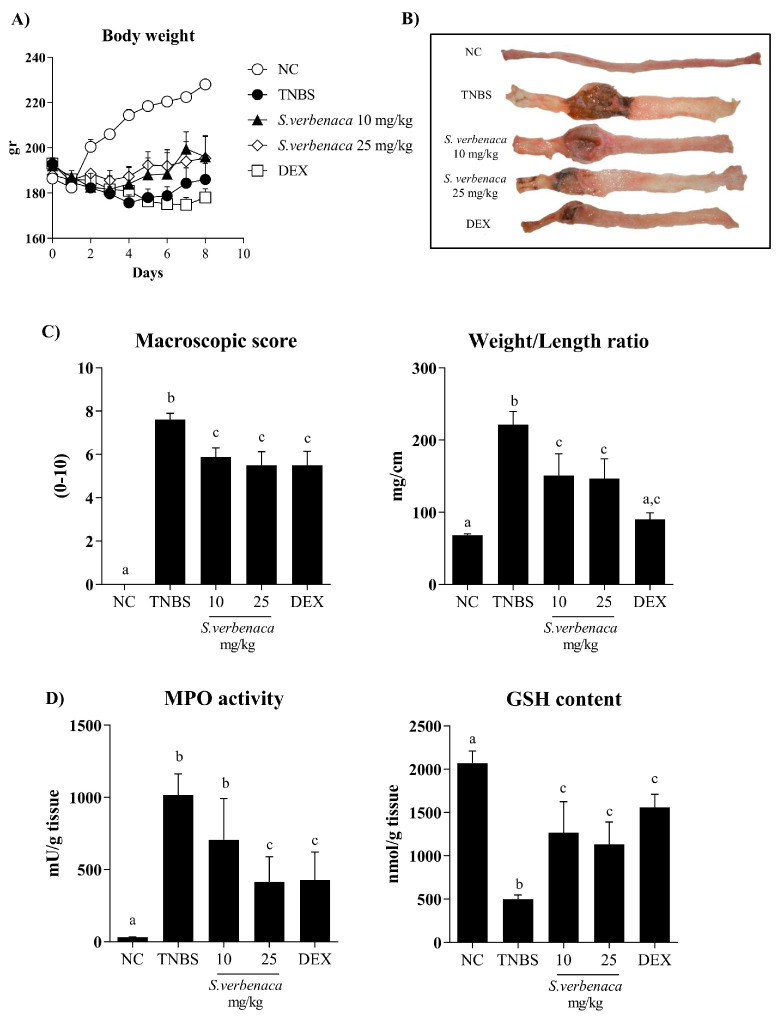
Effects of different doses (10–25 mg/kg) of *S. verbenaca* extract and dexamethasone (DEX) (1.2 mg/kg) on (**A**) animal body weight, (**B**) macroscopic colon damages (after longitudinal opening), (**C**) macroscopic damage score, weight/length ratio, and (**D**) myeloperoxidase (MPO) activity and glutathione (GSH) content in non-colitic (NC) and TNBS-induced colitic rats. Data are expressed as means ± SEM (n = 10). Non-parametric data (score) are expressed as the median (range) and were analyzed using the Kruskal–Wallis test. Groups with different letters statistically differ (*p* < 0.05).

Experimental colitis was induced by the intrarectal application of TNBS to rats, a haptenizing agent that triggers an exacerbated immunological response associated with the development of the inflammatory process in the colon. During the experiment, colitic rats from the control group showed reduced food intake and body weight loss (Figure 4A), together with the presence of bloody diarrhea. Once the rats were sacrificed, macroscopic examination of the colonic specimens displayed signs indicative of acute inflammation and necrosis of the colonic tissue (Figure 4B). Additionally, adhesions to adjacent organs were observed.

In addition, the intestinal inflammatory process in the untreated control group was characterized by a marked increase in colonic weight/length ratio (a reliable indicator of tissue edema and inflammation) in comparison with non-colitic rats (Figure 4C). Administration of *S. verbenaca* extract, at doses of 10 and 25 mg/kg, significantly reduced the macroscopic damage score (*p* < 0.05), thus attenuating the severity and extension of the colonic injury (Figure 4C). Similarly, colitic rats receiving the extract showed a lower colonic weight/length ratio in comparison with untreated controls (Figure 4C). The administration of the glucocorticoid dexamethasone (1.2 mg/kg) to colitic rats also resulted in a significant amelioration of the weight/length ratio as well as in the colonic damage score when compared with untreated TNBS control animals (Figure 4C). 

The intestinal anti-inflammatory effects of *S. verbenaca* were also supported by biochemical evidence. Thus, TNBS administration increased colonic MPO activity (Figure 4D), a reliable indicator of neutrophil infiltration in the inflamed intestine. Interestingly, and confirming the results obtained macroscopically, the *S. verbenaca*-treated group showed a decrease in colonic MPO activity, although this was statistically different only at the highest dose assayed in comparison to the untreated TNBS-control group (Figure 4D). Additionally, colonic inflammation-associated oxidative stress was evidenced by a reduced colonic glutathione content, a potent antioxidant peptide, which was markedly increased in colitic animals treated with the extract or dexamethasone (Figure 4D). 

**Figure 5:**
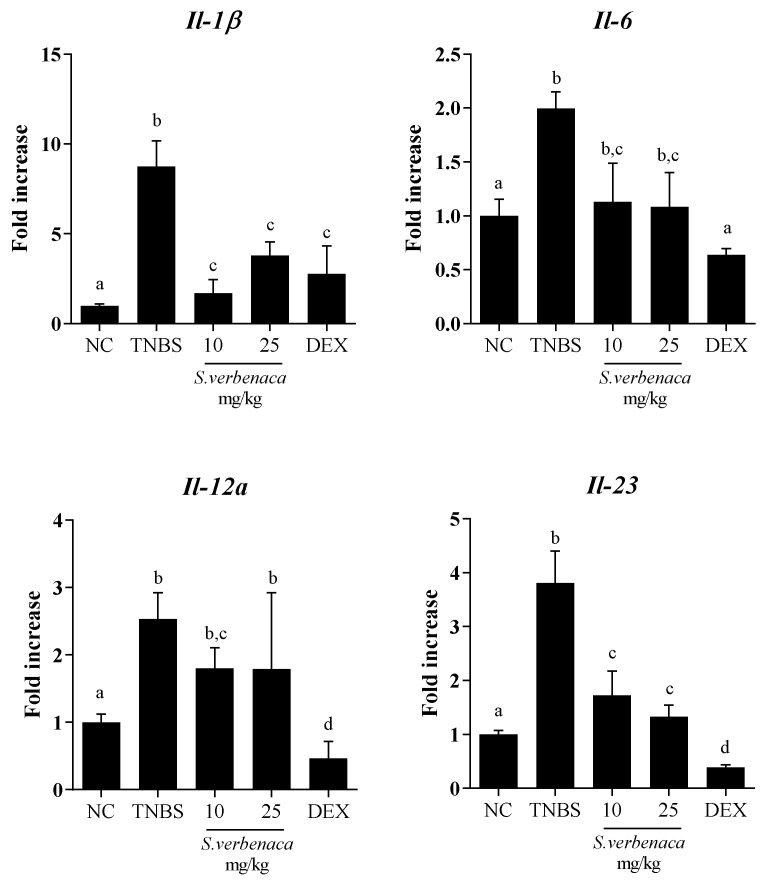
Effects of different doses (10–25 mg/kg) of *S. verbenaca* extract and dexamethasone (DEX) (1.2 mg/kg) on colonic gene expression of *Il-1β*, *Il-6*, *Il-12a*, and *Il-23* non-colitic (NC) and TNBS-induced colitic rats. Data are expressed as means ± SEM (n = 10). Groups with different letters statistically differ (*p* < 0.05).

Likewise, colonic inflammatory status was also characterized by significant increased mRNA expression of various pro-inflammatory markers, including *Il-1β*, *Il-6*, *Il-12a*, and *Il-23*, in the colonic tissue from untreated TNBS colitic animals compared with non-colitic rats (Figure 5). The *S. verbenaca*-treated group showed a marked downregulation in the mRNA expression of *Il-1β*, *Il-6*, *Il-12a* and *Il-23* (*p* < 0.05 vs. colitic control group), thus confirming the amelioration in the colonic inflammatory status exerted by the extract in this experimental model of colitis. Similarly, colitic rats treated with dexamethasone also displayed significantly reduced expression levels of all these cytokines (Figure 5).

**Figure 6:**
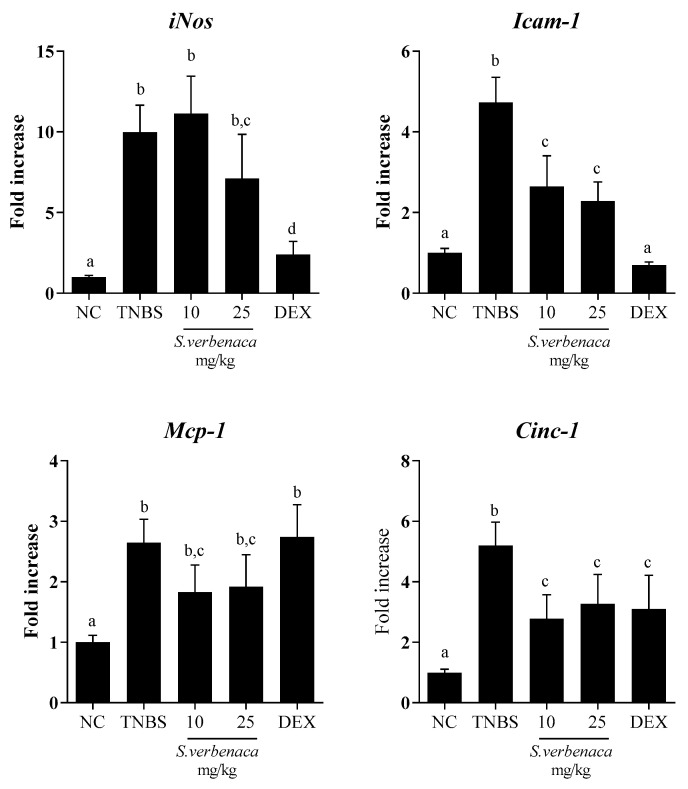
Effects of different doses (10–25 mg/kg) of *S. verbenaca* extract and dexamethasone (DEX) (1.2 mg/kg) on colonic gene expression of *iNos*, *Icam-1*, *Mcp-1*, and *Cinc-1* in non-colitic (NC) and TNBS-induced colitic rats. Data are expressed as means ± SEM (n = 10). Groups with different letters statistically differ (*p* < 0.05).

In addition, the colonic inflammatory process was also characterized by the increased expression of the inducible enzyme *iNos* when compared to the untreated colitis control group. The administration of *S. verbenaca* extract (25 mg/kg) or dexamethasone to colitic rats significantly decreased the elevated colonic expression of *iNos* (Figure 6). This reduction may lead to a decreased production of nitric oxide (NO), potentially preventing the reported adverse effects associated with high NO production in the inflamed colonic tissue [24]. The colonic injury induced by TNBS administration was also evidenced by the increased expressions of the intercellular adhesion molecule-1 (*Icam-1*) as well as the chemokines monocyte chemotactic protein-1 (*Mcp-1*) and cytokine-induced neutrophil chemoattractant-1 (*Cinc-1*) (Figure 6). The expression of these markers was notably reduced by the administration of the extract of *S. verbenaca*, at both doses assayed, as well as by the glucocorticoid. 

**Figure 7:**
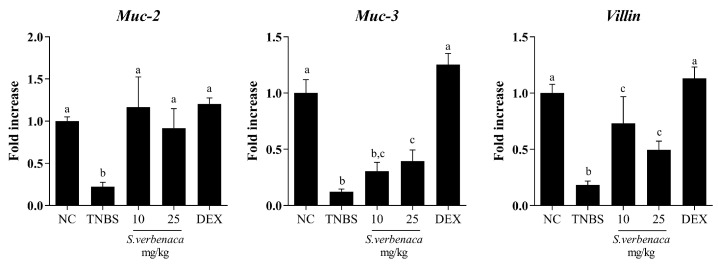
Effects of different doses (10–25 mg/kg) of *S. verbenaca* extract and dexamethasone (DEX) (1.2 mg/kg) on markers of intestinal barrier integrity *Muc-2*, *Muc-3*, and *Villin* in non-colitic (NC) and TNBS-induced colitic rats. Data are expressed as means ± SEM (n = 10). Groups with different letters statistically differ (*p* < 0.05).

Similarly, when the colonic barrier integrity was evaluated in control colitic rats without treatment, an impaired colonic expression of the mucins *Muc-2*, *Muc-3*, and *Villin* was observed, indicating a defect in the colonic permeability (Figure 7). Of note, both the extract and dexamethasone significantly increased the expression of these mediators, thus resulting in the amelioration of the intestinal barrier dysfunction typically observed in colitic rats (Figure 7).

## 4. Discussion

Despite the largely unknown etiology of IBD, accumulated experimental and clinical data suggest that the altered immunological functions resulting from the interplay between genetic predisposition and certain environmental factors can facilitate the mucosal inflammation of the gastrointestinal tract in these chronic conditions [25,26]. 

Nowadays, many drugs used in IBD treatment have positive efficacy, but high price, inconvenient dosing regimen, as well as severity and frequency of adverse effects limit their long-term use [7,27]. For this reason, additional research approaches to IBD therapy are emerging. Among these, the use of alternative and/or complementary treatments, such as herbal remedies, is increasing constantly [28,29]. However, well planned scientific studies focusing on the safety or efficacy of these natural products are limited. With this purpose, the present study aimed to evaluate the immunomodulatory and anti-inflammatory effects of a well-characterized extract from *S. verbenaca* in the TNBS model of experimental rat colitis, which shows some resemblance to the main manifestations of human IBD [16]. Firstly, *S. verbenaca* administration to colitic rats significantly attenuated the symptoms that characterize the disease; both doses of the extract resulted in the reduction of body weight loss as well as the prevention of diarrhea and rectal bleeding. The beneficial effects of *S. verbenaca* were also shown macroscopically, with a decrease of mucosal edema and hemorrhagic ulcerations caused by TNBS instillation, and then confirmed biochemically, with an amelioration of several inflammatory markers implicated in the pathogenesis of IBD. Emerging evidence suggests oxidative stress as a main player in these inflammatory processes. Reactive oxygen species (ROS), derived from activated phagocytic cells and myeloperoxidase activity, are increased in both UC and CD. High ROS levels in mucosal cells participate in the induction of inflammatory and immune responses, which could directly or indirectly cause the gut mucosal barrier dysfunction that orchestrates the colitis process [30,31]. The treatment of colitic rats with the extract of *S. verbenaca* showed a partial inhibition of MPO activity in the inflamed colon compared to untreated colitic rats, suggesting its ability to reduce neutrophil infiltration into the colonic mucosa [32]. In this regard, the extract reduced the colonic expression of critical markers involved in chemotaxis, such as *Cinc-1* and *Mcp-1*, thus collaborating in the attenuation of leukocyte infiltration into the tissue injury [33]. Moreover, the activation of innate and/or adaptive immune responses in IBD involves an increased expression of other mediators, including adhesion molecules [34]. Interestingly, treatment with *S. verbenaca* demonstrated the capacity to modify the increased expression of *Icam-1* in both in vivo and in vitro experimental conditions. Moreover, the in vitro experiments conducted in the CMT-93 cell line showed that the extract was able to downregulate the expression of *Mip-2*, a chemokine that affects neutrophil recruitment and activation [35], thus justifying the results shown in vivo.

It is well known that neutrophils constitute an important source of free radicals, thus contributing to oxidative stress associated with the intestinal inflammatory process. The impact of the extract on neutrophil infiltration can account for reduced free radical production. Supporting this, the administration of the extract to colitic animals counteracted the depletion of the colonic glutathione, thus preserving the colonic tissue from the inflammation-derived oxidative damage in experimental colitis [36]. Moreover, the antioxidant properties ascribed to the extract of *S. verbenaca* and evidenced in the different in vitro assays in the present study can also ameliorate the oxidative stress in the inflamed colon, preserving it from the damage induced after TNBS colonic administration to rats.

Furthermore, the imbalance between pro-inflammatory and anti-inflammatory cytokines in the colonic tissue is crucial in the onset and perpetuation of the disease in intestinal inflammation [37]. Different studies have reported that inflamed colonic mucosa from CD and UC patients are characterized by the increased expression of pro-inflammatory cytokines such as *Il-6*, *Il-1β*, *Il-12a*, *Il-23*, and *Tnf-α* [38,39,40]. In the present study, the intestinal anti-inflammatory effect of *S. verbenaca* on TNBS-induced colitis was linked to a reduced expression of pro-inflammatory mediators. This gives evidence for the improvement of the altered colonic immune response that occurs in the TNBS-colitis model. Notably, the immunomodulatory properties of the extract were also revealed in vitro since it decreased the expression and/or release of key pro-inflammatory cytokines such as IL-6, and TNF-α in LPS-stimulated CMT-93 murine epithelial cells. 

Overall, all these beneficial properties exhibited by the extract can be attributed to the presence of various bioactive compounds that can act simultaneously on different targets of the inflammatory response. Indeed, the results obtained by the DPPH assay revealed that the extract displays antioxidant properties (16.8%), probably correlated to its high content of caffeic acid and phenolic derivatives such as flavonoids. Of note, these bioactive compounds are potent antioxidants that present interesting anti-inflammatory activity [41,42]. In fact, in vitro and in vivo studies have demonstrated that salvianolic acid (another active constituent of *S. verbenaca*) can regulate the expression of antioxidant enzymes by scavenging oxygen free radicals, thus providing hydrogen atoms and reducing the production of oxygen-containing non-radicals and oxygen free radicals [43]. 

Similarly, caffeic acid derivatives can scavenge nitric oxide as well as modulate *iNOS* expression [41], while flavonoids can inhibit transcription factors or regulatory enzymes essential for controlling inflammatory mediators. Regarding the latter, previous studies have reported that flavonoids such as quercitrin, rutin, glabridin, naringenin, chrysin, and cardamonin can reduce the severity of experimental-induced colitis and ameliorate the histological damage by reducing cytokine release in the colonic tissue [44,45,46]. Some of the mechanisms proposed to support these beneficial effects have been related to its ability to inhibit cellular pathways, including nuclear factor-κB (NF-κB), mitogen-activated protein kinase (MAPK), and STAT activation [47,48,49], which actively contribute to the pathology of these intestinal conditions [50,51]. 

Moreover, many of these compounds have been also associated with the preservation of the epithelial barrier function [42,52]. It is well known that dysregulation of the epithelial barrier is a salient feature of IBD [53,54]. A defective epithelial barrier with compromised adherence and tight junctions can lead to an excessive access of luminal agents into the intestinal tissue, thus facilitating the induction of an exacerbated inflammatory response [55]. These changes also compromise epithelial barrier functions, which, if impaired, can cause inappropriate secretion of intestinal fluid and electrolytes, in association with a reduced intestinal absorptive capacity, thus resulting in abdominal cramps and diarrhea, frequently observed in IBD patients with active disease. 

Phytochemical compounds such as naringenin [45] and salvianolic acid [56] have demonstrated their ability to enhance epithelial barrier permeability in colitis. This improvement was observed through the preservation of the intestinal tight junction barrier function and structure, both of which were found to be compromised after DSS administration. Similarly, in our study, TNBS-induced colitis resulted in a reduction in the expression of *Muc-2* and *Muc-3*, which are key constituents of the colonic mucus layer [57,58], and *villin*, a bioactive peptide involved in epithelial repair and regeneration [59]. The administration of the extract significantly reversed the reduced expression of these proteins involved in maintaining an adequate colonic barrier function, thus preserving the mucus-secreting layer covering the epithelium and acting as a physical barrier against luminal content translocation. This beneficial effect can probably be also ascribed to the presence of flavonoids in the extract, given the reported ability to reduce intraluminal fluid accumulation, enhance intestinal motility, and inhibit muscle contractility, as evidenced in different experimental studies [60,61].

## 5. Conclusions

*S. verbenaca* exerted intestinal anti-inflammatory effects in the TNBS-induced colitis in rats. The antioxidant properties linked to its phenolic components seem to play a pivotal role. Additionally, other mechanisms can also mediate the observed beneficial effects in experimental colitis, including their capacity to downregulate the expression and production of various proinflammatory mediators, such as cytokines and chemokines, as well as to enhance intestinal barrier integrity. These results suggested that *S. verbenaca* extract may represent a potential complementary candidate in the management of gastrointestinal disorders.

## Figures and Tables

**Table 1:** Criteria for assessment of macroscopic colonic damage in rat TNBS-induced colitis.

Score	Criteria
0	No damage
1	Hyperemia, no ulcers
2	Linear ulcer with no significant inflammation
3	Linear ulcer with inflammation at one site
4	Two or more sites of ulceration/inflammation
5	Two or more major sites of ulceration and inflammation or one site of ulceration/inflammation extending along the length of the colon
6–10	If damage covers along the length of the colon, the score is increased by 1 for each additional centimeter of involvement

**Table 2:** RT-qPCR primer sequences.

Gene	Organism	Sequence 5′–3′	Annealing T °C	Accesion Number
*Gapdh*	Mouse/Rat	FW: CCATCACCATCTTCCAGGAG RV: CCTGCTTCACCACCTTCTTG	60	NM_001289726.1
*Il6*	Mouse	FW: TAGTCCTTCCTACCCCAATTTCC RV: TTGGTCCTTAGCCACTCCTTCC	60	NM_031168.2
*Icam-1*	Mouse	FW: CAGTCCGCTGTGCTTTGAGA RV: CGGAAACGAATACACGGTGAT	62	NC_000075.7
*Mip-2*	Mouse	FW: CAGTGAGCTGCGCTGTCCAATG RV: CAGTTAGCCTTGCCTTTGTTCAG	57	NC_000071.7
*Muc2*	Mouse	FW: GCAGTCCTCAGTGGCACCTC RV: CACCGTGGGGCTACTGGAGAG	60	NC_000073.7
*Tnfα*	Mouse	FW: AACTAGTGGTGCCAGCCGAT RV: CTTCACAGAGCAATGACTCC	60	NM_001278601.1
*Cinc-1*	Rat	FW: GGCAGGGATTCACTTCAAGA RV: GCCATCGGTGCAATCTATCT	60	NC_051349.1
*Icam-1*	Rat	FW: AGGTATCCATCCATCCCACA RV: AGTGTCTCATTGCCACGGAG	60	NC_051343.1
*Il1β*	Rat	FW: GATCTTTGAAGAAGAGCCCG RV: AACTATGTCCCGACCATTGC	59	NC_051338.1
*Il6*	Rat	FW: CTTCCCTACTTCACAAGTC RV: CTCCATTAGGAGAGCATTG	60	NC_051339.1
*Il12a*	Rat	FW: ACGCTACCTCCTCTTCTTG RV: ATGTCGTCCGTGGTCTTC	60	NC_051337.1
*Il17*	Rat	FW: TGGACTCTGAGCCGCAATGAGG RV: GACGCATGGCGGACAATAGAGG	60	NC_051344.1
*Il23*	Rat	FW: ATCCAGTGTGGTGATGGTTGTG RV: TGTCCGAGTCCAGCAGGTG	60	NC_051342.1
*iNos*	Rat	FW: AAGAGACGCACAGGCAGAGG RV: AGCAGGCACACGCAATGAT	60	NC_051345.1
*Mcp-1*	Rat	FW: CACTATGCAGGTCTCTGTCACG RV: CTGGTCACTTCTACAGAAGTGC	60	NC_051345.1
*Muc-2*	Rat	FW: ACCACCATTACCACCACCTCAG RV: CGATCACCACCATTGCCACTG	60	NC_051336.1
*Muc-3*	Rat	FW: CACAAAGGCAAGAGTCCAGA RV: ACTGTGCTTGGTGCTGAATG	60	NC_051347.1
*Villin*	Rat	FW: TGTGGAACTGGCAGGGAG RV: GGGGTGGGTCTTGAGGTATT	59	NC_051344.1

**Table 3:** Chemical composition of *S. verbenaca* extract.

Peak	RT	Measured *m*/*z*	Molecular Formula	Proposed Compounds	Main Fragments
1	1.01	195.0538	C_6_H_12_O_7_	Gluconic acid	NF
2	1.14	133.0165	C_4_H_6_O_5_	Malic acid	NF
3	1.28	191.0226	C_6_H_8_O_7_	Citric acid	NF
4	1.58	96.9610	-	Sulfate	NF
5	1.73	96.9610	-	Sulfate	NF
6	2.20	191.0559	C_7_H_12_O_6_	Quinic acid	NF
7	2.39	395.0965	C_18_H_20_O_10_	Acetylisobiflorin	179
8	2.74	315.0723	C_13_H_16_O_9_	Gentisoyl glucoside	152, 108
9	2.85	551.1058	C_24_H_24_O_15_	Phloroscorbinol hexaacetate	NF
10	3.19	153.0188	C_7_H_6_O_4_	Dihydroxybenzoic acid	109
11	3.38	181.0502	C_9_H_10_O_4_	Dihydrocaffeic acid isomer 1	135
12	3.67	175.0608	C_7_H_12_O_5_	Isopropylmalic acid	NF
13	4.35	391.0667	C_18_H_16_O_10_	Pentahydroxy trimethoxy flavone isomer 1	347, 303
14	4.55	325.0927	C_15_H_18_O_8_	p-Coumaric acid glucoside	163
15	4.72	391.0661	C_18_H_16_O_10_	Pentahydroxy trimethoxy flavone isomer 2	347, 303
16	5.66	475.0886	C_22_H_20_O_12_	Chrysoeriol glucuronide	299, 227
17	6.22	475.1833	C_21_H_32_O_12_	Cistanoside E	179, 135
18	6.32	181.0504	C_9_H_10_O_4_	Dihydrocaffeic acid isomer 2	NF
19	6.80	179.0346	C_9_H_8_O_4_	Caffeic acid	135
20	7.34	593.1158	C_30_H_26_O_13_	O-coumaroyl orientin	NF
21	7.65	639.1199	C_27_H_28_O_18_	Quercetin glucosyl-glucuronide isomer 1	463, 300
22	7.89	405.0834	C_19_H_18_O_10_	Glucopyranosyl-trihydroxy-9H-xanthen-9-one	273, 317, 387
23	8.12	639.1157	C_27_H_28_O_18_	Quercetin glucosyl-glucuronide isomer 2	299
24	8.20	589.0826	C_19_H_26_O_21_	Unknown 1	
25	8.28	783.1637	C_33_H_36_O_22_	Quercetin glucoside derivative	607, 505, 463, 300, 545
26	8.48	353.0876	C_16_H_18_O_9_	Caffeoyl quinic acid	191, 179
27	8.63	347.0777	C_17_H_16_O_8_	Dihydroxy tetramethoxyxanthone	NF
28	8.78	655.1288	C_31_H_28_O_16_	Acetylated flavonol glycoside isomer 1	285, 461, 447
29	8.86	329.0774	C_14_H_18_O_9_	Flavonol derivative	285
30	8.92	655.1292	C_31_H_28_O_16_	Acetylated flavonol glycoside isomer 2	285, 461
31	8.96	655.1299	C_31_H_28_O_16_	Acetylated flavonol glycoside isomer 3	285, 461, 447
32	9.02	637.1058	C_27_H_26_O_18_	Luteolin diglucuronide	351, 285
33	9.10	571.1082	C_27_H_24_O_14_	Yunnaneic acid E isomer 1	197, 285, 135, 527, 241, 329, 439
34	9.23	571.1083	C_27_H_24_O_14_	Yunnaneic acid E isomer 2	197, 135, 285, 347, 527, 241, 483, 439
35	9.29	539.1189	C_27_H_24_O_12_	Yunnaneic acid D	297, 179, 197, 161, 135, 359
36	9.35	415.1953	C_20_H_32_O_9_	Yunnaneic acid derivative 1	329, 179, 161, 297
37	9.47	597.1263	C_29_H_26_O_14_	Yunnaneic acid F	197, 135, 329, 179
38	9.63	309.0616	C_14_H_14_O_8_	Feruloylmalic acid	193, 134
39	9.71	555.1157	C_27_H_24_O_13_	Salvianolic acid K isomer 1	197, 135, 329, 179
40	9.76	585.1273	C_28_H_26_O_14_	Naringenin digalloylglucopyranoside isomer 1	271
41	9.79	585.1244	C_28_H_26_O_14_	Naringenin digalloylglucopyranoside isomer 2	271
42	9.84	585.1221	C_28_H_26_O_14_	Naringenin digalloylglucopyranoside isomer 3	271
43	9.87	461.0740	C_21_H_18_O_12_	Luteolin glucuronide	285
44	9.95	361.0938	C_18_H_18_O_8_	Crotepoxide	273, 241
45	10.03	527.1208	C_26_H_24_O_12_	Yunnaneic acid derivative 2 isomer 1	135, 197, 179, 285
46	10.15	719.1629	C_36_H_32_O_16_	Sagerinic acid	359, 161, 197, 179
47	10.24	527.1198	C_26_H_24_O_12_	Yunnaneic acid derivative 2 isomer 2	285, 197, 135, 241, 179, 439
48	10.33	717.1479	C_33_H_34_O_18_	Salvianolic acid L	359, 161, 197
49	10.49	511.1293	C_26_H_24_O_11_	Salvianolic acid A hydrate	269, 197, 135, 179
50	10.58	343.0924	C_22_H_16_O_4_	Unknown 2	
51	10.64	555.1153	C_27_H_24_O_13_	Salvianolic acid K isomer 2	359, 161, 135, 493, 401, 537
52	10.74	711.3987	C_37_H_60_O_13_	Hydroxytormentic acid derivative isomer 1	503
53	10.87	533.1302	C_25_H_26_O_13_	Yunnaneic acid derivative 3 isomer 1	197
54	10.93	541.1365	C_27_H_26_O_12_	Yunnaneic acid derivative 4	197, 509, 135, 179
55	10.95	533.1315	C_25_H_26_O_13_	Yunnaneic acid derivative 3 isomer 2	197
56	10.99	537.1053	C_27_H_22_O_12_	Lithospermic acid A	295, 161, 359, 135, 197, 493
57	11.06	651.2319	C_31_H_40_O_15_	Martynoside	175, 475
58	11.17	373.0936	C_19_H_18_O_8_	Methyl rosmarinate isomer 1	197, 161
59	11.21	373.0917	C_19_H_18_O_8_	Methyl rosmarinate isomer 2	135, 175, 197
60	11.38	711.3966	C_37_H_60_O_13_	Hydroxytormentic acid derivative isomer 2	503
61	11.43	481.1125	C_25_H_22_O_10_	Silybin	301, 283
62	11.62	493.1144	C_26_H_22_O_10_	Salvianolic acid A	295
63	11.71	479.0992	C_25_H_20_O_10_	Dehydrosilybin	299, 281
64	11.76	491.0985	C_26_H_20_O_10_	Salvianolic acid C	293
65	11.99	327.2151	C_18_H_32_O_5_	Trihydroxyoctadecadienoic acid	NF
66	12.19	695.4023	C_37_H_60_O_12_	Unknown 3	
67	12.47	329.2330	C_18_H_34_O_5_	Pinellic acid	NF
68	12.57	523.1242	C_27_H_24_O_11_	Salvianolic acid derivative	135, 179, 491, 359
69	12.77	345.1717	C_20_H_26_O_5_	Rosmanol isomer 1	NF
70	13.56	307.1909	C_18_H_28_O_4_	Unknown 4	
71	14.20	345.1702	C_20_H_26_O_5_	Rosmanol isomer 2	301, 283
72	14.32	311.2206	C_18_H_32_O_4_	Octadecenedioic acid	NF
73	14.74	331.1918	C_20_H_28_O_4_	Carnosic acid	287, 244
74	14.99	721.3674	C_34_H_58_O_16_	Palmitoleic-linolenicglucoside	397, 277, 415, 235
75	15.80	293.2116	C_18_H_30_O_3_	Hydroxylinolenic acid isomer 1	NF
76	15.82	293.2119	C_18_H_30_O_3_	Hydroxylinolenic acid isomer 2	NF
77	16.39	295.2276	C_18_H_32_O_3_	Hydroxylinoleic acid	NF
78	16.68	455.3168	C_29_H_44_O_4_	Diosgenin acetate	NF
79	17.22	471.3479	C_30_H_48_O_4_	Maslinic acid isomer 1	NF
80	17.36	471.3485	C_30_H_48_O_4_	Maslinic acid isomer 2	NF

**Table 4:** Total phenolic content and antioxidant capacity of *S. verbenaca*.

Method	Value
Folin–Ciocalteu (mg GAE/g d.e.)	166 ± 4
FRAP (mmol eq. FeSO4/g d.e.)	2.6 ± 0.1
TEAC (mmol eq. Trolox/g d.e.)	0.73 ± 0.02
ORAC (mmol eq. Trolox/g d.e.)	0.92 ± 0.03

GAE = gallic acid equivalents; d.e. = dry extract; eq. = equivalent.

## Data Availability

The data presented in this study are available on request from the corresponding author.
